# Novel *RP1* mutations and a recurrent *BBS1* variant explain the co-existence of two distinct retinal phenotypes in the same pedigree

**DOI:** 10.1186/s12863-014-0143-2

**Published:** 2014-12-14

**Authors:** Cristina Méndez-Vidal, Nereida Bravo-Gil, María González-del Pozo, Alicia Vela-Boza, Joaquín Dopazo, Salud Borrego, Guillermo Antiñolo

**Affiliations:** Department of Genetics, Reproduction and Fetal Medicine, Institute of Biomedicine of Seville, University Hospital Virgen del Rocío/CSIC/University of Seville, Avenida Manuel Siurot s/n, 41013 Seville, Spain; Centro de Investigación Biomédica en Red de Enfermedades Raras (CIBERER), Seville, Spain; Genomics and Bioinformatics Platform of Andalusia (GBPA), Seville, Spain; Computational Genomics Department, Centro de Investigación Príncipe Felipe (CIPF), Valencia, Spain

**Keywords:** Bardet-Biedl syndrome, *BBS1*, Inherited retinal dystrophies, Next-generation sequencing, Retinitis pigmentosa, *RP1*

## Abstract

**Background:**

Molecular diagnosis of Inherited Retinal Dystrophies (IRD) has long been challenging due to the extensive clinical and genetic heterogeneity present in this group of disorders. Here, we describe the clinical application of an integrated next-generation sequencing approach to determine the underlying genetic defects in a Spanish family with a provisional clinical diagnosis of autosomal recessive Retinitis Pigmentosa (arRP).

**Results:**

Exome sequencing of the index patient resulted in the identification of the homozygous *BBS1* p.M390R mutation. Sanger sequencing of additional members of the family showed lack of co-segregation of the p.M390R variant in some individuals. Clinical reanalysis indicated co-ocurrence of two different phenotypes in the same family: Bardet-Biedl syndrome in the individual harboring the *BBS1* mutation and non-syndromic arRP in extended family members. To identify possible causative mutations underlying arRP, we conducted disease-targeted gene sequencing using a panel of 26 IRD genes. The in-house custom panel was validated using 18 DNA samples known to harbor mutations in relevant genes. All variants were redetected, indicating a high mutation detection rate. This approach allowed the identification of two novel heterozygous null mutations in *RP1* (c.4582_4585delATCA; p.I1528Vfs*10 and c.5962dupA; p.I1988Nfs*3) which co-segregated with the disease in arRP patients. Additionally, a mutational screening in 96 patients of our cohort with genetically unresolved IRD revealed the presence of the c.5962dupA mutation in one unrelated family.

**Conclusions:**

The combination of molecular findings for *RP1* and *BBS1* genes through exome and gene panel sequencing enabled us to explain the co-existence of two different retinal phenotypes in a family. The identification of two novel variants in *RP1* suggests that the use of panels containing the prevalent genes of a particular population, together with an optimized data analysis pipeline, is an efficient and cost-effective approach that can be reliably implemented into the routine diagnostic process of diverse inherited retinal disorders. Moreover, the identification of these novel variants in two unrelated families supports the relatively high prevalence of *RP1* mutations in Spanish population and the role of private mutations for commonly mutated genes, while extending the mutational spectrum of *RP1*.

**Electronic supplementary material:**

The online version of this article (doi:10.1186/s12863-014-0143-2) contains supplementary material, which is available to authorized users.

## Background

Genetic testing of inherited retinal dystrophies (IRDs) is a highly complex and time-consuming process. More than 200 genes have been associated with this heterogeneous group of disorders (RetNet, https://sph.uth.edu/retnet/home.htm), characterized by the primary dysfunction or loss of photoreceptors leading to progressive visual impairment and blindness. Further complicating the molecular diagnosis of IRDs, clinical diagnosis can also be challenging due to IRD clinical features overlap. The identification of causative mutations is important for several reasons, among them, it allows geneticists to give an adequate genetic counseling to the families, to establish genotype-phenotype correlations that predictably improve the prognosis of the disease, and to monitor the efficacy and safety of new therapeutic options.

Retinitis Pigmentosa (RP; MIM **#**268000) represents the most prevalent clinical subtype of IRDs, affecting 1 in ~4,000 individuals. The clinical course of RP involves progressive degeneration of rods and subsequent involvement of cones as the disease progresses. To date, more than 60 genes have been associated with RP, being *EYS* the most prevalent gene worldwide [1–3]. Although RP is usually manifested as a non-syndromic disease, it can also be associated with a wide variety of extraocular symptoms constituting syndromic forms of RP, being Bardet-Biedl syndrome one of the most common (BBS; MIM #209900). BBS is a model ciliopathy with high genetic and clinical heterogeneity, generally inherited as an autosomal recessive trait [4]. The estimated prevalence in Europe is approximately 1/125,000 [5]. This disorder is characterized by a combination of clinical symptoms including obesity, RP, post-axial polydactyly, polycystic kidneys and learning disabilities, some of which may appear many years after the onset of the disease. Clinical expression is variable but many patients manifest most of the clinical symptoms during the course of illness. The number of causative genes is relatively high with 18 different genes associated so far (Ret Net; accessed 2014 Mar 1), including *BBS1* (MIM #209901), that accounts for approximately 40 % of BBS families.

One of the first RP-associated genes to be discovered was *RP1* (MIM #603937). This gene is located on chromosome 8q12 and consists of 4 exons [6]. It encodes an oxygen-regulated photoreceptor protein with two microtubule-binding, doublecortin-like domains (DCX) at the N-terminus involved in microtubule stabilization. Additionally, the C-terminal region has been implicated in the binding of RP1 to proteins destined for the outer segment [7]. Human RP1 specifically localizes to the connecting cilia of rod and cone photoreceptors also suggesting a role in the transport of proteins between the inner and outer segments and/or in maintenance of cilial structure [8]. The vast majority of pathogenic mutations in this gene are truncating variants clustered in exon 4 (Human Gene Mutation Database, HGMD, http://www.hgmd.cf.ac.uk/ac/index.php; accessed 2014 Mar 24). Mutations in *RP1* can cause both autosomal dominant (adRP) [9,10] or autosomal recessive RP (arRP) [11–13], and although several mechanisms have been suggested to explain the mutational mechanisms in *RP1* leading to dominant or recessive RP [14], no clear models have been proposed.

The recently introduced next-generation sequencing (NGS) technologies have greatly facilitated the molecular diagnosis of IRD, in particular, in poorly defined or misclassified clinical cases [15]. At present, targeted analysis of disease-specific candidate genes is most suitable for diagnostic applications as it facilitates functional interpretation of sequence variations and overcomes limitations in computational analysis [16]. In this study, we describe the successful application of an integrated NGS strategy to provide an accurate clinical and molecular diagnosis of concurrent BBS and arRP in a family provisionally diagnosed of arRP. The definitive clinical diagnosis of the index patient was made based on clinical re-examination and sequencing data generated through whole-exome sequencing to identify the *BBS1* p.M390R mutation [17] responsible for the BBS phenotype. Likewise, disease-targeted gene sequencing resulted in the identification of two novel truncating mutations in RP1 (p.I1528Vfs*10 and p.I1988Nfs*) in extended family members affected of arRP.

## Methods

### Subjects and clinical assessment

**Figure 1:**
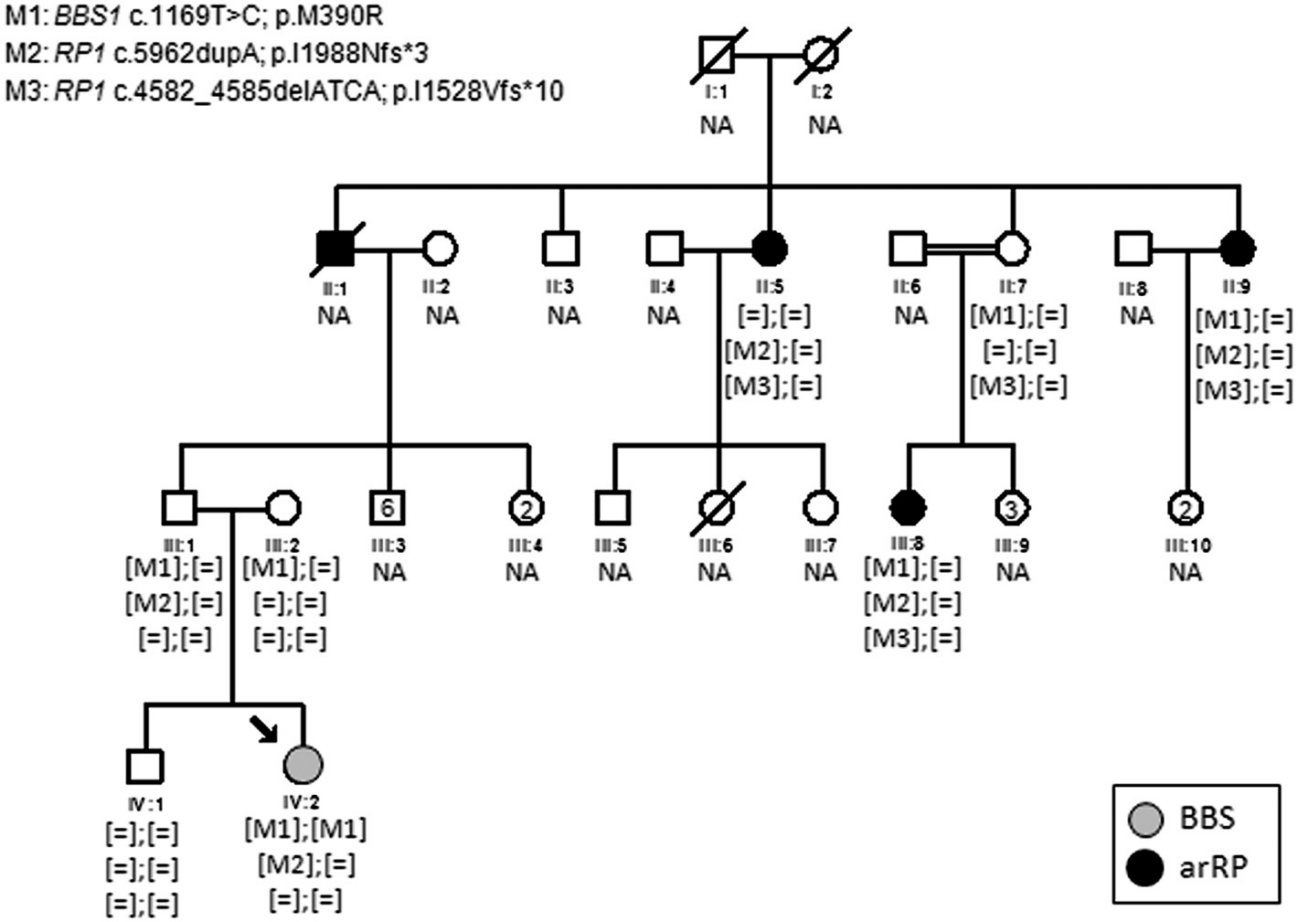
**Family cosegregation analysis.** Pedigree showing segregation of the *BBS1* and *RP1* mutations. [M];[M]: Homozygous, [M];[=]: Heterozygous, [=];[=]: Wild type, NA: Not available.

Our study involved one Spanish family (RP368) native to a small rural area with five affected members provisionally diagnosed of arRP and four available healthy individuals (Figure 1) recruited from the Ophthalmology Department and derived to the Genetic, Reproduction and Fetal Medicine Department. Clinical diagnosis of arRP was established by ophthalmological examination as described elsewhere [18]. For validation purposes of the IRD panel, DNA samples of 18 IRD patients displaying different degrees of retinal degeneration and with known causal mutations (Table 1) were included in the study. In addition, a group of 200 matching control individuals and 96 IRD patients of our cohort without a molecular diagnosis were also recruited. Written informed consent was obtained from all participants. Also, expressed consent was signed by all participants for the publication of their clinical data. The study was reviewed and approved by the ethics committees of our institution and performed in accordance with the tenets of the Declaration of Helsinki [19]. Peripheral blood was extracted for genomic DNA isolation from leukocytes using the MagNA Pure LC system (Roche, Indianapolis, IN) according to the manufacturer’s protocol.

**Table 1:** **IRD panel test samples**

**Samples**	**Phenotype**	**Gene**	**Chr location**	**Nt change**	**Prot change**	**Status**	**Detection method**	**Cvg mut**
**1**	XLRP	*RPGR*	X:38145845	c.2405-2406delAG	p.E802Gfs*32	HEMI	WES	26
**2**	ARRP	*CRB1*	1:197403836	c.2843G > A	p.C948Y	HOM	Reseq	125
**3**	ARRP	*USH2A*	1:215955412	c.10712C > T	p.T3571M	HET	Asper	160
		*USH2A*	1:216420460	c.2276G > T	p.C759F	HET	Asper	70
**4**	USHER	*CDH23*	10:73553078	c.6393delC	p.I2132Sfs*11	HOM	Asper	176
**5**	ARRP	*MERKT*	2:112751826	c.1297-2A > G	Splicing	HOM	Reseq	39
**6**	ARRP	*RDH12*	14:68196055	c.806_810del5	p.A269Gfs*2	HOM	Reseq	136
**7**	ARRP	*CNGA1*	4:47954625	c.301C > T	p.R101*	HOM	Reseq	19
**8**	ADRP	*RP1*	8:55538471	c.2029C > T	p.R677*	HET	Asper	247
**9**	ADRP	*PRPF3*	1:150316692	c.1481C > T	p.T494M	HET	Asper	35
**10**	XLRP	*RP2*	X:46713107	c.299dupT	p.F101Vfs*23	HEMI	Sanger	24
**11**	STGD	*ABCA4*	1:94506901	c.3386G > T	p. R1129L	HET	Asper	24
		*ABCA4*	1:94508434	c.3210_3211insGT	p.S1071Vfs*14	HET	Asper	24
**12**	ARRP	*NR2E3*	15:72105913	c.932G > A	p.R311Q	HOM	Reseq	28
		*RDH12*	14:68195950	c.701G > A	p.R234H	HET	Reseq	51
**13**	ARRP	*RHO*	3:129252539	c.1025C > T	p.T342M	HOM	Reseq	29
**14**	ADRP	*PRPH2*	6:42672285	c.646C > T	p.P216S	HET	Asper	45
**15**	USHER	*MYO7A*	11:76867944	c.626C > A	p.S210*	HOM	Asper	16
**16**	ADRP	*RHO*	3:129251107	c.544G > A	p.G182S	HET	Reseq	31
**17**	ARRP	*EYS*	6:64776240	c.6714delT	p.I2239Sfs*17	HOM	Sanger	13
**18**	ARRP	*CERKL*	2:182423344	c.769C > T	p.Arg257*	HOM	Asper	73

WES: Whole Exome Sequencing; Reseq: Custom genome resequencing microarray; Asper: commercially available microarray analysis (Asper Biotech); Sanger: Sanger sequencing.

DNA samples from individuals III:1, III:2, IV:1, and IV:2 were processed for WES while DNA samples from 18 patients with previously identified mutations and from individual II:5 were processed for gene panel sequencing.

### Previous molecular genetic analysis

The index patient was first analyzed and excluded for known mutations in arRP genes by applying commercially available microarray analysis (Asper Biotech, Tartu, Estonia), a custom genome resequencing microarray [20] and by direct sequencing of *EYS* [2]. Causative mutations of the IRD panel validation samples were previously identified by different strategies (Table 1).

### Whole-exome sequencing and data analysis

Library preparation and exome capture were performed using NimbleGen SeqCap EZ Exome libraries V3 (Roche NimbleGen, Inc., Madison, WI, USA) and sequenced on a SOLiD 5500xl platform. These procedures and the analysis of data from deep sequencing were carried out as previously described [18,21].

### Design of the capture IRD panel

We developed a capture panel of 26 retinal disease genes commonly mutated in our population (Table 2). Coding exons plus 25 bp of intronic flanking sequence of targeted genes and their genomic coordinates were identified using UCSC Genome Browser (http://genome.ucsc.edu/) and submitted to Roche NimbleGen (Madison, WI) to generate a SeqCap EZ Choice Library of hybridization probes. The probes covered a total of 510 exons and the entire custom design spanned 118,238 bp. The final capture size was 248,054 bp.

**Table 2:** **List of genes included in the capture IRD panel**

**Gene**	**Chr location**	**NCBI reference sequence**	**Exons**	**Pathology**
*ABCA4*	1:94458391-94586688	NM_000350.2	50	ADRP, ARRP, ARCRD, ARMD, STGD
*CDH23*	10:73156691-73575702	NM_022124.5	69	USH, Deafness alone or syndromic
*CERKL*	2:182401403-182545392	NM_201548.4	14	ARRP, ARCRD
*CNGA1*	4:47937994-48018689	NM_001142564.1	10	ARRP
*CRB1*	1:197170592-197447585	NM_201253.2	12	ARRP, ARLCA
*EYS*	6:64429876-66417118	NM_001142800.1	43	ARRP
*FSCN2*	17:79495422-79504156	NM_012418.3	5	ADRP, ADMD
*MERTK*	2:112656056-112787138	NM_006343.2	19	ARRP
*MYO7A*	11:76839310-76926284	NM_000260.3	49	USH, Deafness alone or syndromic
*NR2E3*	15:72084977-72110559	NM_016346.3	8	ADRP, ARRP, ARESCS
*PDE6B*	4:619373-664571	NM_000283.3	22	ARRP, ADCSNB
*PROM1*	4:15964699-16086001	NM_006017.2	28	ARRP, ADCRD, ADMD
*PRPF3*	1:150293925-150325671	NM_004698.2	16	ADRP
*PRPF31*	19:54618837-54635140	NM_015629.3	14	ADRP
*PRPH2*	6:42664340-42690312	NM_000322.4	3	ADRP, ADMD, ADCRD and digenic
*RDH12*	14:68168603-68201169	NM_152443.2	9	ADRP, ARLCA
*RHO*	3:129247483-129254012	NM_000539.3	5	ADRP, ARRP, ADCSNB
*RLBP1*	15:89753098-89764922	NM_000326.4	9	ARRP
*RP1*	8:55471729-55682531	NM_006269.1	4	ADRP, ARRP
*RP2*	X:46696375-46741793	NM_006915.2	5	XLRP
*RPE65*	1:68894505-68915642	NM_000329.2	14	ARRP, ARLCA
*RPGR*	X:38128424-38186817	NM_001034853.1	19	XLRP, XLCRD, XLMD
*SAG*	2:234216309-234255701	NM_000541.4	16	ARRP, ARCSNB
*TULP1*	6:35465651-35480715	NM_003322.4	15	ARRP, ARLCA
*USH1G*	17:72912176-72919351	NM_173477.4	3	USH
*USH2A*	1:215796236-216596738	NM_206933.2	73	ARRP, USH

RP: Retinitis pigmentosa; AD: Autosomal dominant; AR: Autosomal recessive; XL: X-linked; LCA: Leber congenital amaurosis; CRD: Cone or cone-rod dystrophy; MD: Macular degeneration; CSNB: Congenital stationary night blindness; USH: Usher syndrome; STGD: Stargardt disease; ESCS: Enhanced S-cone syndrome.

### DNA library preparation and targeted sequencing

Library preparation was performed according to the manufacturer’s protocol (GS FLX Titanium Rapid Library Preparation Method Manual, January 2010 version). Briefly, 500 ng of input genomic DNA was fragmented by nebulization resulting in a majority of fragments between 600-900 bp. Fragments were end repaired, ligated to the provided adaptors and eluted in water following small fragments removal using AMPure Beads (Beckman Coulter, Agencourt, Beverly, MA). The library was amplified for 12 cycles by pre-capture ligation-mediated PCR (LM-PCR) using FastStart High Fidelity PCR System (Roche, Basel, Switzerland) and specific primers for the adaptors. After purification, 1 μg of LM-PCR product was hybridized to the custom designed SeqCap EZ Library of biotinylated probes for 72 h at 47°C. Captured DNA fragments were purified with streptavidin-conjugated magnetic beads (Invitrogen, Carlsbad, CA, USA) and washed. Amplification was performed for 15 cycles by post-capture LM-PCR using FastStart High Fidelity PCR System. The final concentration of each captured library was measured with Quant-iT PicoGreen dsDNA Assay Kit (Invitrogen, Carlsbad, CA) and diluted to 10^6^ molecules/μl. Emulsion PCR was performed according to the manufacturer’s instructions (emPCR Amplification Method Manual-Lib-L, GS Junior Titanium Series, March 2012). To perform the emPCR an input of 0.7 molecules per bead was chosen. After enrichment, about 500,000 beads were sequenced on 454 Roche GS Junior sequencer according to the manufacturer’s protocol (Sequencing Method Manual GS Junior, Titanium Series, March 2012).

### Bioinformatics analysis

**Figure 2:**
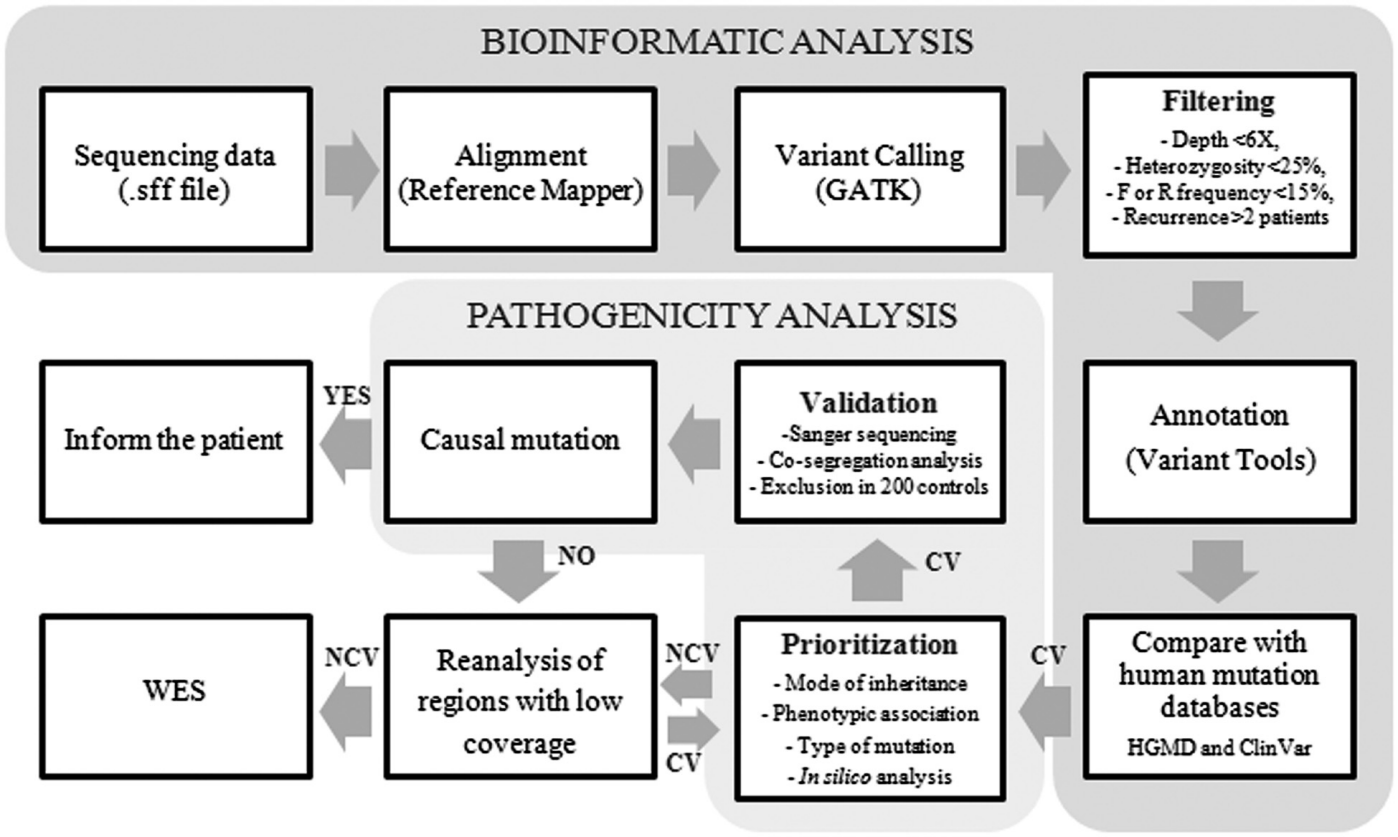
**Pipeline designed for data analysis.** Bioinformatic analysis including mapping, calling, filtering, and annotation of variants, followed by a pathogenicity analysis in which the candidate variants (CV) are prioritized and validated with the aim of finding the causal mutation and inform the patient. A reanalysis of regions with low coverage and WES will be conducted when no candidate variants (NCV) are identified.

An analysis pipeline was developed to identify pathogenic mutations (Figure 2). After duplicates removal, reads were aligned against the human genome reference (hg19) using the GS Reference Mapper software (Roche, version 2.7). Improperly mapped reads were filtered out with the SAMtools package, which was also used to generate, sort and index BAM files. To analyze the coverage and the percentage of reads on target we used the BEDtools package. Variant calling was performed with the software GATK (Genome Analysis Toolkit, version 1.4) [22] to detect SNVs, while indels were called with FreeBayes software (version 0.9.10) (https://github.com/ekg/freebayes). To avoid artifacts, variants with a coverage <6X, a percentage of total reads supporting the variant allele <25%, and variants with a disequilibrium (<15%) between number of forward and reverse sequences were removed. In addition, an in-house developed database of common variations in our cohort was use to eliminate those variants that appeared in more than two patients. Sequence variants annotation was performed using Variant Tools [23]. Annotated variants located in non-coding regions outside of the splice sites, and/or present in the National Center for Biotechnology Information (NCBI) Single Nucleotide Polymorphism database (dbSNP, http://www.ncbi.nlm.nih.gov/SNP/) and 1000 Genomes project (http://www.1000genomes.org/) database, with a MAF higher than 0.01 were discarded. The remaining variants were compared with human mutation databases such as HGMD and ClinVar (http://www.ncbi.nlm.nih.gov/clinvar/) to detect known disease-associated mutations, using the TEAM tool (http://team.babelomics.org) with a definition of the panel described in Table 2. Sequence variants were further prioritized according to inheritance patterns and type of mutations using the BiERapp tool, generating a list of candidate causal mutations (http://bierapp.babelomics.org).

### Verification and determination of the pathogenicity of variants

Predicted disease-causing variants were confirmed by Sanger sequencing using specific primers designed by Primer3 software (http://bioinfo.ut.ee/primer3-0.4.0/) and co-segregated in available family members DNA samples. Novel variants were subsequently screened in 200 healthy matched control subjects by Sanger sequencing and in Exome Variant Server (http://evs.gs.washington.edu/EVS/). The pathogenicity of novel missense substitutions was evaluated using Polymorphism Phenotyping v2 (PolyPhen-2, http://genetics.bwh.harvard.edu/pph2/) and Sorting Intolerant From Tolerant (SIFT, (http://sift.bii.a-star.edu.sg) scores. The correct name of the variation according to the Human Genome Variation Society (http://www.hgvs.org) nomenclature guidelines was checked using Mutalyzer (http://www.LOVD.nl/mutalyzer).

### Microsatellite marker analysis

Microsatellite marker analysis was carried out in available family members DNA samples of the arRP branch. For this purpose, a total of five microsatellite markers flanking *RP1* were selected from UCSC Genome Browser. PCR amplification using genomic DNA samples and primers closely flanking the region containing microsatellite repeats were used for analysis (Additional file 1). PCR products were genotyped using an ABI-3730 sequencer (Applied Biosystems, USA), and analyzed by GeneMapper v.4.0 software (Applied Biosystems).

### Mutational screening of *RP1* in additional IRD patients

To detect additional cases carrying the novel *RP1* mutations, we performed Sanger sequencing in 96 IRD patients of our cohort without a genetic diagnosis. This study group consisted of 75 cases clinically diagnosed of arRP, 3 of adRP, 3 of cone-rod dystrophies, 6 of Leber Congenital Amaurosis, and 9 of Stargardt disease.

## Results

### WES and clinical refinements

The index patient (IV:2) (Figure 1) had received a provisional diagnosis of arRP based on previous family history (II:1, II:5, II:9 and III:8) with clinical manifestations typical of a severe form of RP characterized by early age of onset and a rapid progression of the disease, leading to a total blindness before age 30 in most patients. The first symptom of the index patient was night blindness at age of six, followed by decreased visual acuity, reduced visual field, dyschromatopsia and photophobia. The fundus examination showed signs of RP such as bone spicule pigmentation and attenuation of the retinal vessels.

In order to identify causal mutations, we sequenced the exomes of the index patient, one healthy sibling (IV:1) and both unaffected parents (III:1 and III:2). This allowed the identification of the known homozygous p.M390R mutation in the BBS-associated gene *BBS1*, previously reported by Mykytyn et al, [17]. Lack of segregation of this variant in extended family members prompted us to clinically reevaluate the entire family focusing on the identification of extraocular symptoms associated with BBS. We found that the index patient showed characteristic BBS features including obesity, learning disabilities and postaxial polydactyly, resulting in a clinical reclassification of this family branch from arRP to BBS. In contrast, fundus examination in the rest of affected members showed typical signs of RP with pale optic nerve disc, retinal vessels attenuation and bone spicule pigmentation in the periphery, confirming a diagnosis of non-syndromic arRP in individuals II:5, II:9 and III:8. Therefore, the causal mutation of this family branch remained to be discovered.

Given that individual II:1 was also affected of arRP, we hypothesized that patient III:1 must be an obligate carrier of one of the arRP mutations. To assess this possibility, a screening of heterozygous mutations in known RP genes present in the WES data of individual III:1 was carried out as a first approach to detect the cause of the disease in the arRP members, but no candidates were found.

### Validation of the customized IRD panel

The genetic cause of arRP was studied by sequencing 26 IRD genes. In order to assess the reliability of our capture panel as a diagnostic tool, we tested 18 samples with known causative variants on exons or bordering sequences in 17 retinal disease genes included in the panel. A total of 21 mutations were checked, 6 indels and 15 SNVs.

#### Data quality

**Figure 3:**
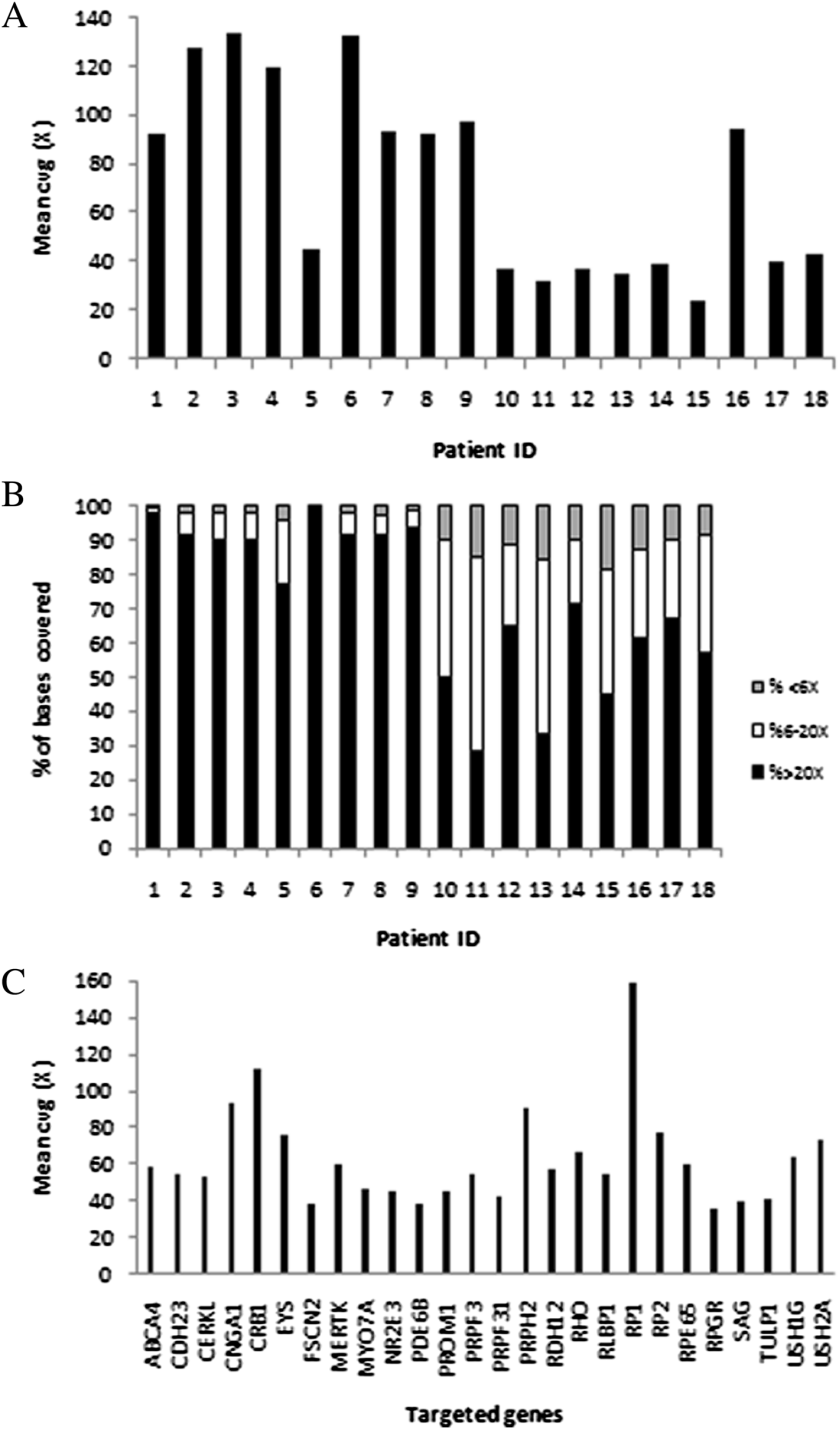
**Coverage analysis of 454 pyrosequencing data of the 18 samples used for IRD panel validation. (A)** Mean coverage of the targeted regions for each individual. **(B)** Percentage of targeted positions covered less than 6x (gray), between 6x and 20x (white), and more than 20x (black), for each individual. **(C)** Mean coverage for each targeted gene.

For each sample, gene panel sequencing generated an average of data output of 56 Mb and 134,656 reads per run with a mean length of 416 bp. The mean coverage obtained for the 18 validation samples was 71x (Figure 3A), with 72% of reads covered >20x and 93% >6x (Figure 3B). On average, 98% of targeted bases were covered indicating that the coverage was sufficiently high for sensitive detection of variants. The specific coverage for the 21 checked mutations is shown in Table 1. Furthermore, the mean coverage for the 26 targeted genes ranged between 36x (*RPGR*) and 159x (*RP1*) (Figure 3C). The mean percentage of reads mapped on target was 55%, while up to 70% of sequences overlapped the targeted region. However, only 4 samples showed a percentage of reads on target lower than 50% (samples #10, #11, #13, and #18), achieving most samples a ratio greater than 60%.

#### Detection, filtering, and verification of variants

On average, targeted sequencing of validation samples revealed 2,318 variants per patient (616 SNVs and 1,702 indels). The filtering of variants was performed as detailed in Materials and Methods and resulted in a mean of 48 variants per sample. After comparing with human mutation databases HGMD and ClinVar, a mean of 2 reported variants were identified for each sample, among which the causative mutation was found in 14 cases. The prioritizing step left 1-2 candidate variants per sample to be validated. After validation, 22 variants were considered possible causative mutations, of which 21 matched the causal mutations previously identified, and only one additional novel mutation in *RP1* in sample #8 was found (c.3956A > G, p.E1319G). Therefore, all variants were properly redetected using this NGS approach and verification of underrepresented NGS regions by Sanger sequencing was not required in this study.

### Identification of pathogenic mutations

**Figure 4:**
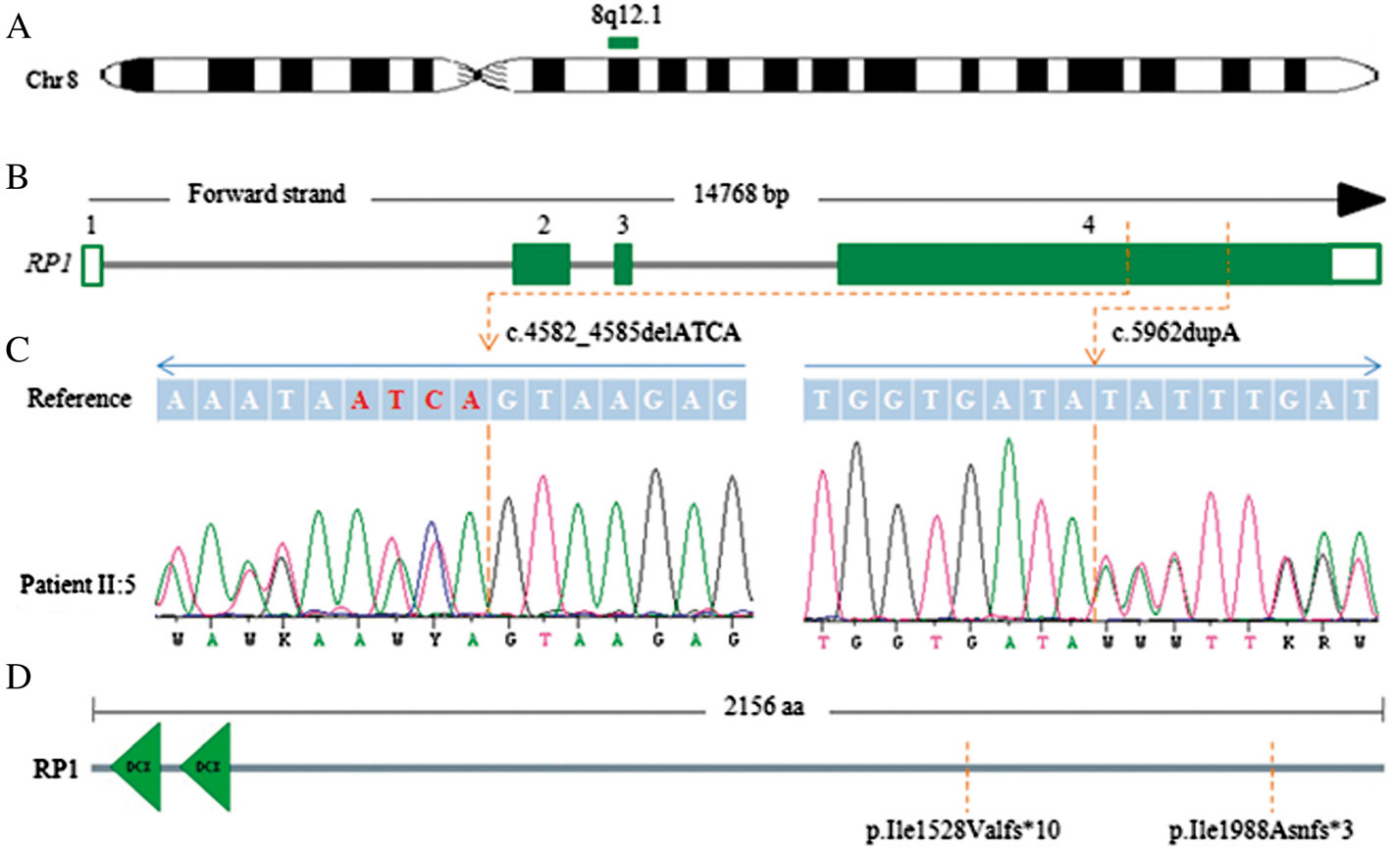
**Schematic representation of the novel** ***RP1*** **mutations. (A)** Overview of chromosome 8. *RP1* is mapped on region 8q12.1 (green bar). **(B)** Gene structure of *RP1* gene containing 4 exons. The position of identified variants is stated with a broken line. Coding exons are shown as filled boxes while unfilled boxes reflect UTRs. **(C)** Electropherograms of patient II:5 showing the heterozygous mutations in exon 4 of *RP1* gene. **(D)** RP1 protein representation with two doublecortin domains (DCX) marked in green, and the location of variants indicated by a broken line.

To identify causative mutations of non-syndromic arRP in family RP368, the specific IRD panel was used to predict potential pathogenic variants in patient II:5. This sample showed a mean coverage of 32x, with the 99.5% of targeted bases covered and a percentage of reads on target of 50%. Application of the previously described automatic variant calling, filtering, and annotation pipeline of the capture sequencing data from this patient detected two heterozygous variants located on exon 4 of *RP1* (c.4582_4585delATCA; p.Ile1528Valfs*10 and c.5962dupA; p.Ile1988Asnfs*3) (Figure 4), with an specific coverage of 111x and 87x, respectively. The two mutations were confirmed by Sanger sequencing and cosegregation in available arRP family members. Both mutations were novel and had never been reported in public variant databases such as dbSNP, EVS or 1000 genomes database. Additionally, we did not detect such changes in 200 control individuals. These novel frameshift mutations (p.Ile1528Valfs*10 and p.Ile1988Asnfs*3) resulted in premature termination codons, causing a truncated RP1 protein in both cases. Thus, the compound heterozygosity for these mutations was proposed as the most probable disease-causing mechanism in the arRP members.

To discard the possibility that the two mutations were located on the same allele, we performed a linkage analysis using five microsatellite markers flanking the *RP1* gene on individuals II:5, II:7, II:9 y III:8. The haplotypes confirmed that each mutation is found in different alleles (Additional file 2).

### Mutational screening of *RP1* mutations in unresolved IRD cases

Direct full sequencing of the two novel *RP1* mutations in 96 IRD patients without a molecular diagnosis allowed the detection of the c.5962dupA mutation in homozygosity in one additional family of our cohort (Additional file 3). This family was not related to family RP368. Clinically, patient with homozygous mutation c.5962dupA showed signs of typical early onset RP and resembles arRP affected members of family RP368.

## Discussion

Clinical and genetic complexity of IRD makes the accurate diagnosis of some cases only accessible by NGS approaches. Here, we followed a strategy that combines exome and disease-targeted gene panel sequencing to assist in the molecular diagnosis of a family affected of both BBS and RP. Whole exome sequencing allowed the identification of the *BBS1* p.M390R mutation as the BBS-causing variant while the genetic cause of non-syndromic arRP was assessed by gene panel sequencing.

Compared to exome sequencing, targeting fewer genes has clear advantages including the analysis of more patient samples per instrument cycle, a greater depth of coverage, a simplified data interpretation and less restrictive ethics. Validation of our disease-targeted gene panel was critical to ensure high quality interpretation of clinical evidences. All types of variants were detected by this platform indicating a high specificity of mutation detection. Of note, the identification of the c.2405_2406delAG variant located in the hot spot exon ORF15 of *RPGR*, a domain that usually remains problematic for sequencing, indicated that the enrichment technology produced suitable calls for use in clinical laboratories.

Analysis of our NGS data also resulted in the identification of definite candidate variants for each test sample with the exception of validation sample #8, harboring not only the causal adRP mutation p.R677* in *RP1*, but also an additional variant (p.E1319G). The fact that the vast majority of pathogenic mutations in *RP1* result in premature stop codons (nonsense and frameshift mutations) indicate that the most probable adRP-causative mutations in validation sample #8 is p.R667* and not p.E1319G, which might be secondary to the disease and simply reflect the high frequency of carriers harboring heterozygous mutant alleles of IRD genes in the general population [24]. Additionally, the p.R677* mutation has been reported as the most frequently pathogenic *RP1* mutation [14,25] with a location consistent with a dominant inheritance pattern [12].

Studies performed after this panel design have shown that, of all solved cases in our cohort, 98% of families carry mutations in one of the genes included in the panel while 2% of cases harbor mutations in other genes (unpublished data), indicating that, as expected, we primarily find novel mutations in prevalent genes in our population. Therefore, although we did not aimed at evaluating the diagnostic yield in this study, we can expect that a large proportion of pending cases may be solved using this panel which is remarkable given the small target size compared to other IRD panels [15,26–29]. Nonetheless, we are aware that to increase the diagnostic yield of the panel, the analysis of other types of variants such as large CNVs or structural variants must be considered [30]. Also, genes newly found to be mutated in our cohort as well as non-coding regions with previously reported deep-intronic pathogenic mutations [31–33] must be included.

The implementation of this tool for the molecular diagnosis of arRP individuals resulted in the reliable identification of two novel *RP1* mutations (c.4582_4585delATCA; p.Ile1528Valfs*10 and c.5962dupA; p.Ile1988Asnfs*3). Family segregation showed indeed the presence of the variant c.5962dupA in heterozygous state in individual III:1. However, this variant was filtered out during the re-analysis of the WES data of heterozygous variants in RP genes in this individual due to low coverage (4x), supporting the use of gene panels to increase the coverage of certain target regions.

Screening of our cohort for these mutations led to the molecular diagnosis of an unrelated arRP family carrying the c.5962dupA variant in homozygosis. The presence of this variant in two unrelated families of our cohort seems to indicate the recurrent nature of this novel variant in Spanish population. These data are in agreement with a recent report showing that the contribution of *RP1* to arRP in the Spanish population may have been underestimated [13].

It has been previously hypothesized that the specific location of mutations in *RP1* may be an important factor in determining their functional effect [7]. Given that both frameshift mutations found in this study are located in the second half of exon 4, the mutated mRNA would not be theoretically susceptible to the nonsense-mediated decay (NMD) pathway [34], resulting in prematurely truncated proteins. It has been proposed that while larger disruptions of RP1 are often associated with adRP due to deleterious effects of the truncated proteins, the lack of only the most terminal portion results in a loss of RP1 function, causing arRP [7,12]. This would explain that the two novel detected mutations are pathogenic in compound heterozygosity or homozygosity, while heterozygous carriers of only one of them are asymptomatic. Besides their location, the type of mutation appears to be also important to the etiopathogenesis of RP associated with *RP1* mutations. Both families diagnosed in this study showed similar clinical features with severe RP, characterized by early age of onset and a rapid progression of the disease. These clinical characteristics are consistent with previous studies [11,13] describing that patients with arRP due to *RP1* mutations show severe RP and present equivalent phenotypes in different families.

Previous reports have shown other cases of apparent clinical heterogeneity due to independent segregation of two different retinal phenotypes secondary to mutations in two different genes [35,36]. Interestingly, other cases of concurrent retinal disorders have been explained by the presence of p.M390R and mutations in the arRP-associated genes *C2ORF71* and *RP1* [37,38]. In this study we further highlight how isolated population or consanguinity could increase the risk factor of developing diverse IRDs in the same family. Consequently, it is important to consider the existence of different genetic causes in the same family when clinical manifestations and genetic data do not correlate.

## Conclusions

The genetic findings for *RP1* and *BBS1* genes revealed co-occurrence of two distinct retinal phenotypes resulting in the accurate diagnosis of a poorly defined clinical case. The detection of two novel pathogenic *RP1* variants p.Ile1528Valfs*10 and p.Ile1988Asnfs*3 suggests that the use of IRD panels of a small size, limited to prevalent genes of a particular population, together with an optimized data analysis pipeline, is an efficient and cost-effective approach for the diagnosis of diverse retinal disorders. Moreover, the implementation of this panel as a diagnostic tool can be useful to determine the prevalence of known IRD genes in our cohort. In fact, the identification of RP1 mutations in two unrelated families supports the relatively high prevalence of this gene in Spanish population.

## Additional files

Additional file 1:
**Microsatellite primers used in the indirect study of** ***RP1*** **segregation.**
Additional file 2:
**Haplotypes of available DNA samples of the arRP family branch using microsatellite markers flanking** ***RP1*** **.** The risk haplotype is shown in black and alleles that do not cosegregate with RP are marked in white. Recombination events implicating the marker D8S532 were observed in patient II:5. Additional file 3:
**Pedigree showing segregation of the mutation c.5962dupA of** ***RP1*** **in available family members. [M];[M]: Homozygous, [M];[=]: Heterozygous; NA: Not available.**
