# Supplementary material for: Novel RP1 mutations and a recurrent BBS1 variant explain the co-existence of two distinct retinal phenotypes in the same pedigree
Source: BMC Genet. 2014 Dec 14;15:143. doi: 10.1186/s12863-014-0143-2 (PMC4271491; doi:10.1186/s12863-014-0143-2)
Supplement: Additional file 1: — Microsatellite primers used in the indirect study of RP1 segregation. [file 12863_2014_143_MOESM1_ESM.pdf]

ADDITIONAL FILES

Additional file 1. Microsatellite primers used in the indirect study of *RP1* segregation.

Microsatellite	Flanking	Forward Primer	Reverse Primer
**D8S285**	*RP1*	GCATCACACAGAATCTTTG	ATGGGTTTATGGCCTTTAC
**D8S260**	*RP1*	AGGCTTGCCAGATAAGGTTG	GCTGAAGGCTGTTCTATGGA
**D8S532**	*RP1*	GCTCAAAGCCTCCAATGAC	GACTTCGTGATCCACCTGC
**D8S1737**	*RP1*	TGTAACACACAGATGCACGC	AAGGATAACTTTGGCTTCGG
**D8S1828**	*RP1*	AGTGCTGTTTTTACTTCTGTACG	GCAAGACTCTGTCTCAGGA
