# Supplementary material for: Novel RP1 mutations and a recurrent BBS1 variant explain the co-existence of two distinct retinal phenotypes in the same pedigree
Source: BMC Genet. 2014 Dec 14;15:143. doi: 10.1186/s12863-014-0143-2 (PMC4271491; doi:10.1186/s12863-014-0143-2)
Supplement: Additional file 2: — Haplotypes of available DNA samples of the arRP family branch using microsatellite markers flanking RP1 . The risk haplotype is shown in black and alleles that do not cosegregate with RP are marked in white. Recombination events implicating the marker D8S532 were observed in patient II:5. [file 12863_2014_143_MOESM2_ESM.pdf]

ADDITIONAL FILES

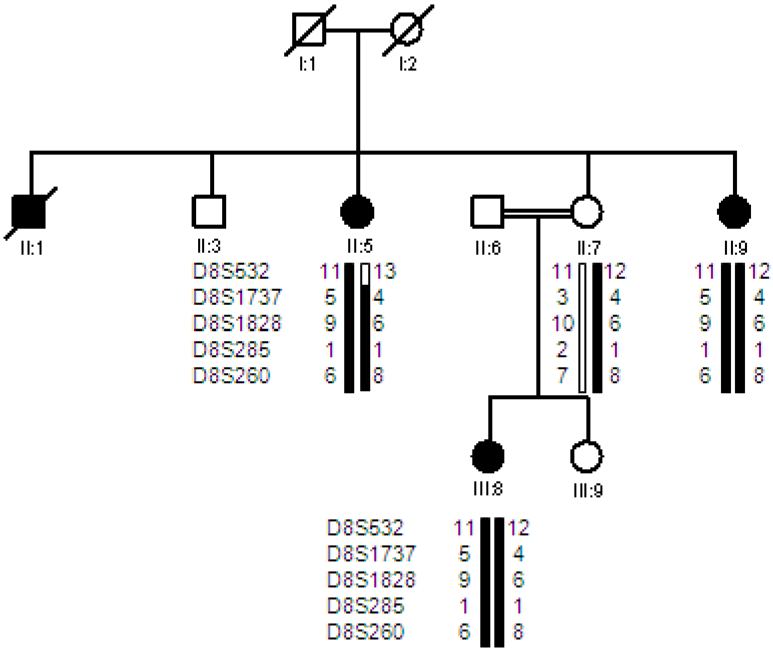

Additional file 2. Haplotypes of available DNA samples of the arRP family branch using microsatellite markers flanking *RP1*. The risk haplotype is shown in black, and alleles that do not cosegregate with RP are marked in white. Recombination events implicating the marker D8S532 were observed in patient II:5.
