# Supplementary material for: Novel RP1 mutations and a recurrent BBS1 variant explain the co-existence of two distinct retinal phenotypes in the same pedigree
Source: BMC Genet. 2014 Dec 14;15:143. doi: 10.1186/s12863-014-0143-2 (PMC4271491; doi:10.1186/s12863-014-0143-2)
Supplement: Additional file 3: — Pedigree showing segregation of the mutation c.5962dupA of RP1 in available family members. [M];[M]: Homozygous, [M];[=]: Heterozygous; NA: Not available. [file 12863_2014_143_MOESM3_ESM.pdf]

ADDITIONAL FILES

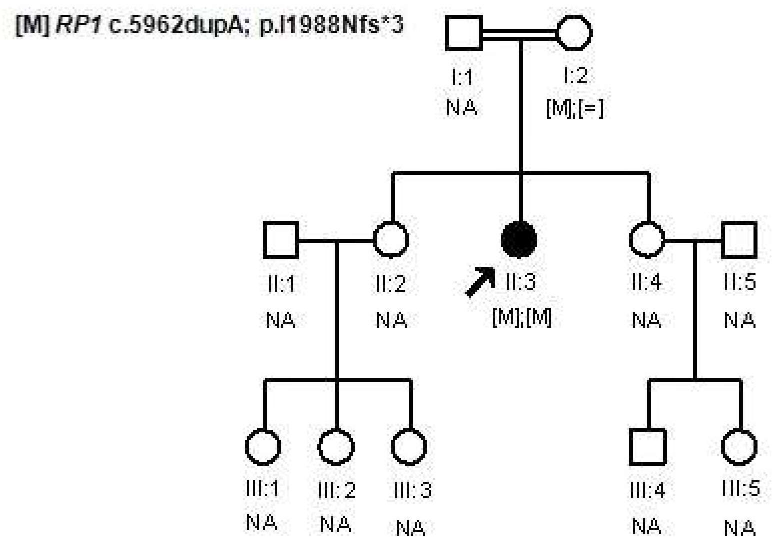

Additional file 3. Pedigree showing segregation of the mutation c.5962dupA of *RP1* in available family members. [M];[M]: Homozygous, [M];[=]: Heterozygous; NA: Not available.
